# The accuracy of reverse genetics systems for SARS‐CoV‐2: Circular polymerase extension reaction versus bacterial artificial chromosome

**DOI:** 10.1111/irv.13109

**Published:** 2023-03-17

**Authors:** Yuri Furusawa, Seiya Yamayoshi, Yoshihiro Kawaoka

**Affiliations:** ^1^ Division of Virology, Institute of Medical Science University of Tokyo Tokyo Japan; ^2^ The Research Center for Global Viral Diseases National Center for Global Health and Medicine Research Institute Tokyo Japan; ^3^ International Research Center for Infectious Diseases, Institute of Medical Science University of Tokyo Tokyo Japan; ^4^ Department of Pathobiological Sciences, School of Veterinary Medicine University of Wisconsin‐Madison Madison Wisconsin USA

**Keywords:** BAC, CPER, reverse genetics, SARS‐CoV‐2

## Abstract

**Background:**

Reverse genetics systems to rescue viruses from modified DNA are useful tools to investigate the molecular mechanisms of viruses. The COVID‐19 pandemic prompted the development of several reverse genetics systems for SARS‐CoV‐2. The circular polymerase extension reaction (CPER) method enables the rapid generation of recombinant SARS‐CoV‐2; however, such PCR‐based approaches could introduce unwanted mutations due to PCR errors.

**Methods:**

To compare the accuracy of CPER and a classic reverse genetics method using bacterial artificial chromosome (BAC), SARS‐CoV‐2 Wuhan/Hu‐1/2019 was generated five times using BAC and five times using CPER. These 10 independent virus stocks were then deep sequencing, and the number of substitutions for which the frequency was greater than 10% was counted.

**Results:**

No nucleotide substitutions with a frequency of greater than 10% were observed in all five independent virus stocks generated by the BAC method. In contrast, three to five unwanted nucleotide substitutions with a frequency of more than 10% were detected in four of the five virus stocks generated by the CPER. Furthermore, four substitutions with frequencies greater than 20% were generated in three virus stocks by using the CPER.

**Conclusions:**

We found that the accuracy of the CPER method is lower than that of the BAC method. Our findings suggest care should be used when employing the CPER method.

## 1 INTRODUCTION

Severe acute respiratory syndrome coronavirus 2 (SARS‐CoV‐2) was identified as a novel human pathogen in China at the end of 2019, and it continues to circulate globally. Since the World Health Organization (WHO) declared a pandemic on March 11, 2020, many variants of concern including omicron have emerged. Reverse genetics is a useful tool with which to study the functional effects of mutations. Since the emergence of SARS‐CoV‐2, several reverse genetics systems for SARS‐CoV‐2 have been reported.^1^, ^2^, ^3^, ^4^, ^5^ One of them, the circular polymerase extension reaction (CPER) method, which is a PCR‐based, bacterium‐free method, is widely used because of its speed and simplicity.^1^, ^2^, ^6^, ^7^, ^8^, ^9^ However, PCR‐based methods come with the risk of PCR errors. In contrast, the reverse genetics system that uses bacterial artificial chromosome (BAC) is a classical method to rescue recombinant viruses possessing a large genome such as coronaviruses and herpes viruses.^10^, ^11^, ^12^ Because BACs are amplified by *Escherichia coli*, the BAC method takes longer to prepare recombinant viruses than PCR‐based methods. However, the BAC method using cloned and sequence‐verified BACs yields recombinant viruses with a very low genetic mixture. To compare the two methods, here, we generated SARS‐CoV‐2 five times using the BAC method and five times using the CPER method and assessed the sequence accuracy by deep sequencing.

## 2 MATERIALS AND METHODS

### 2.1 Cells

VeroE6/TMPRSS2 (JCRB 1819) cells^13^ were propagated in the presence of 1 mg/mL geneticin (G418; Invivogen) and 5 μg/mL plasmocin prophylactic (Invivogen) in Dulbecco's modified Eagle's medium (DMEM) containing 10% fetal calf serum (FCS). Human embryonic kidney 293T (HEK293T) cells were cultured in DMEM supplemented with 10% FCS. VeroE6/TMPRSS2 cells and HEK293T cells were maintained at 37 °C under 5% CO_2_. The cells were regularly tested and confirmed to be negative for mycoplasma contamination by using PCR.

### 2.2 BAC construction

The full‐genome nucleotide sequence of SARS‐CoV‐2 Wuhan/Hu‐1/2019 was assembled into the pBeloBAC11 vector to generate infectious cDNA clones under the control of a cytomegalovirus (CMV) promoter by using Gibson Assembly Master Mix (NEB, E2611) as described previously^14^ with some modifications. Briefly, five fragments (Fa to Fe) covering the full‐length SARS‐CoV‐2 genome with a 30‐bp overlap and restriction sites and linearized pBeloBAC11 (linker fragment) were amplified by PCR using PrimeSTAR GXL DNA Polymerase (TaKaRa), which is a high fidelity PCR enzyme,^15^ and six primer sets (Table 1). The pBeloBAC11 vector was used as a template for PCR to amplify the linker fragment. The artificially synthesized DNA of SARS‐CoV‐2 Wuhan/Hu‐1/2019 was used as a template for PCR to amplify the five fragments, Fa to Fe. First, Fa and Fe were ligated with the linker by using Gibson Assembly Master Mix (NEB) resulting in a BAC containing Fa and Fe. Fb and Fc were subsequently ligated into the BAC digested with FseI and AscI. Finally, Fd was cloned into the BAC digested with FseI and AscI resulting in a BAC carrying the full‐length SARS‐CoV‐2 genome. The constructed BACs were introduced into DH10B *E. Coli* (NEB) by electroporation. Four to six clones were sequenced in each step to select a clone without mutations. The *E. Coli* was amplified at 37°C, and BACs were extracted by using NucleoBond Xtra Maxi (TaKaRa).

**TABLE 1:** Primers used for bacterial artificial chromosome construction.

Fragment	Direction	Sequence (5′ → 3′)
Linker	Forward	TAGCTTCTTAGGAGAATGACAAAAAAAAAAAAAAAAAAAAAAAAAGGGTCGGCATGGCAT
	Reverse	CTGGGAAGGTATAAACCTTTAATACGGTTCACTAAACGAGCTC
Fa	Forward	ATTAAAGGTTTATACCTTCCCAG
	Reverse	AGATTCTCATAAACAAATCCATAAGTTCGTGGCGCGCCGATGGCCGGCCGATGAAATGGTAATTTGTATAGTTTCTAAA
Fb	Forward	TTTAGAAACTATACAAATTACCATTTCATCTTTTAAATGGGATTTAACTG
	Reverse	AAACATTAAAGTTTGCACAATGCAGAATGCATCTGTCATCCAAACAGTTA
Fc	Forward	GCATTCTGCATTGTGCAAACTTTAATGTTTTATTCTCTACAGTGTTCCCA
	Reverse	AGATTCTCATAAACAAATCCATAAGTTCGTGGCGCGCCGATGGCCGGCCTGTTCGTTTAGTTGTTAACAAGAACATCAC
Fd	Forward	GTGATGTTCTTGTTAACAACTAAACGAACAATGTTTGTTTTTCTTGTTTT
	Reverse	AGATTCTCATAAACAAATCCATAAGTTCGTTTATGTGTAATGTAATTTGA
Fe	Forward	ACGAACTTATGGATTTGTTTATGAGAATCTTCACAATTGGAACTGTAACT
	Reverse	TTTTTTTTTTTTTTTTTTTTTTTTTGTCATTCTCCTAAGAAGCTA

### 2.3 CPER

Viruses were rescued by CPER as previously described.^2^ In brief, the artificially synthesized DNA of SARS‐CoV‐2 Wuhan/Hu‐1/2019 was used as a template for PCR. The six DNA fragments covering the full‐length SARS‐CoV‐2 genome and a linker fragment encoding hepatitis delta virus ribozyme, bovine growth hormone polyA signal, and the CMV promoter were amplified by PCR using PrimeSTAR GXL DNA Polymerase, a high fidelity PCR enzyme. The seven DNA fragments were mixed and used for CPER.^2^

### 2.4 SARS‐CoV‐2 rescue

To rescue recombinant SARS‐CoV‐2, BACs or CPER products were transfected into HEK293T cells by using TransIT‐293 (TaKaRa) according to the manufacturer's protocol. At 3 days post‐transfection, the supernatant containing viruses was collected and inoculated onto VeroE6/TMPRSS2 cells for virus propagation, and the supernatant was stored as a virus stock after full cytopathic effect appearance. For the BAC method, the BAC transfection was performed five times independently, and five independent virus stocks were prepared. For the CPER method, PCR amplification of DNA fragments and the CPER and transfection were performed five times independently, and five independent virus stocks were prepared. All experiments with SARS‐CoV‐2 were performed in enhanced biosafety level 3 containment laboratories at the University of Tokyo.

### 2.5 Sequencing analysis

The stock viruses were subjected to next‐generation sequencing using iSeq100 according to a previous report.^16^ The number of substitutions whose frequency was higher than 10% was counted.

## 3 RESULTS AND DISCUSSION

SARS‐CoV‐2 Wuhan/Hu‐1/2019 was generated five times by using BAC and five times using CPER. In this way, we generated 10 independent virus stocks and subjected them to deep sequencing. As shown in Table 2, no nucleotide substitutions with a frequency of greater than 10% were observed in all five independent virus stocks generated by the BAC method. In contrast, 3–5 unwanted nucleotide substitutions were detected in four of the five virus stocks generated by the CPER. Although these substitutions were not dominant, four substitutions with frequencies greater than 20% were observed in three virus stocks. These results clearly demonstrate that reverse genetics by BAC is highly accurate and that the CPER method comes with a high risk of unwanted mutations.

**TABLE 2:** Number of nucleotide substitutions in bacterial artificial chromosome (BAC) and circular polymerase extension reaction (CPER) stocks.

		Number of nucleotide substitutions detected at the indicated frequency		
		10%–20%	20%–30%	More than 30%
BAC	#1	0	0	0
	#2	0	0	0
	#3	0	0	0
	#4	0	0	0
	#5	0	0	0
CPER	#1	0	0	0
	#2	3	1	1
	#3	4	0	1
	#4	3	0	0
	#5	3	1	0

Although the fidelity of the DNA polymerase for PCR has been improving, it is still lower than that of bacterial polymerase. Therefore, unwanted substitutions in rescued SARS‐CoV‐2 have been observed in some previous studies.^1^, ^7^, ^9^ Unwanted substitutions due to PCR errors are introduced randomly and, therefore, their effects on viral characteristics cannot be estimated. This makes it difficult to explore the functions of an amino acid substitution because we want to generate a parental virus and its mutant with only that single amino acid substitution. Such an experiment requires completely identical genome sequences other than at the position of interest. Therefore, such unwanted and uncontrollable mutations might lead to incorrect conclusions that would not be drawn had the viruses been generated by using the BAC method or cloned and sequenced DNA.

The CPER method is a powerful and rapid tool to generate recombinant viruses and has been frequently used in several studies. However, we need to pay close attention to unwanted mutations in stock viruses prepared by the CPER method or other PCR‐based and non‐cloned methods.

## AUTHOR CONTRIBUTIONS

Yuri Furusawa, Seiya Yamayoshi, and Yoshihiro Kawaoka designed the study. Yuri Furusawa and Seiya Yamayoshi performed the experiments and analyzed the data. Yuri Furusawa, Seiya Yamayoshi, and Yoshihiro Kawaoka wrote the manuscript.

## CONFLICT OF INTEREST STATEMENT

All the authors declared no conflicts of interest related to this work.

### PEER REVIEW

The peer review history for this article is available at https://publons.com/publon/10.1111/irv.13109.

## Data Availability

All data supporting the findings of this study are available within the paper and from the corresponding author upon request. There are no restrictions to obtaining access to the primary data.
